# LIPI-4 and *lmo0180* cooperatively regulate flagellar motility and virulence in *Listeria monocytogenes*

**DOI:** 10.3389/fcimb.2026.1915886

**Published:** 2026-09-03

**Authors:** Caixia Liu, Cong Liu, Shengjie Gao, Xun Ma, Cheng Jia, Yue Jiang, Ling Song, Lu Liu, Yatao Qi, Zhongke Yin, Rong Shan, Guangdong Hu, David T. S. Hayman, Jing Wang

**Affiliations:** 1 College of Animal Science and Technology, Shihezi University, Shihezi, China; 2 Molecular Epidemiology and Public Health Laboratory, Hopkirk Research Institute, Massey University, Palmerston North, New Zealand

**Keywords:** biofilm, food-borne disease, gene knockout, listeriosis, motility, pathogenesis, ST87, zoonosis

## Abstract

**Introduction:**

Deletion of *Listeria* pathogenicity island 4 (LIPI-4) in *Listeria monocytogenes* (*L. monocytogenes*) impairs motility, disrupts flagellar assembly, and markedly upregulates the *lmo0180* gene.

**Methods:**

In this study, the Δ*lmo0180* single-gene deletion mutant and ΔLIPI-4/*lmo0180* double-gene deletion mutant were constructed by homologous recombination. Bacterial motility was assessed, and flagellar biogenesis was observed by transmission electron microscopy. The adhesion, invasion, intracellular proliferation, and cell-to-cell spread capacities were compared across strains. Virulence was evaluated in a mouse model, and transcript levels of major virulence factors were quantified by quantitative real-time PCR.

**Results:**

The results showed that deletion of *lmo0180* alone had no detectable effects on bacterial motility, flagellar formation, or virulence-related phenotypes. In contrast, LIPI-4 deletion enhanced biofilm formation and eliminated motility and flagellar assembly. Simultaneous deletion of LIPI-4 and *lmo0180* restored bacterial motility, *flaA* gene expression, and partial restoration of flagellar assembly; however, the double mutant displayed significant reductions in adhesion, invasion, intracellular proliferation, and cell-to-cell spread in host cells. *In vivo* experiments consistently showed that mice infected with the ΔLIPI-4/*lmo0180* mutant exhibited reduced bacterial loads and tissue damage. Compared with the ΔLIPI-4 strain that exhibited elevated transcript levels of PrfA-controlled virulence genes, the ΔLIPI-4/*lmo0180* mutant exhibited downregulation of most such genes.

**Discussion:**

These transcriptional changes may contribute to the attenuated virulence phenotype. Collectively, the study demonstrates that LIPI-4 and *lmo0*180 interact genetically to influence flagellar motility and virulence in *L. monocytogenes*, a finding that provides new insights into the pathogenic mechanisms of *L. monocytogenes*.

## 1 Introduction

As a Gram-positive, rod-shaped, facultatively anaerobic bacterium, *Listeria monocytogenes* (*L. monocytogenes*) is a significant foodborne pathogen that causes listeriosis, a rare but severe invasive disease (Cvetkovic et al., 2023; Ren et al., 2025). Listeriosis has consistently been classified as a major foodborne disease with substantial public health implications for humans and animals worldwide (Olaimat et al., 2018). Listeriosis poses a significant risk to immunocompromised individuals, pregnant women, and newborns, with potential outcomes including meningitis, meningoencephalitis, septicemia, abortion, and mortality rates reaching 20 to 30% (Barbuddhe and Chakraborty, 2009; Scallan et al., 2011).

The identification of *Listeria* pathogenicity island 4 (LIPI-4) began with MLST analysis linking hypervirulent clonal complex 4 (CC4) to human listeriosis (Maury et al., 2016). Whole-genome sequencing then revealed a unique six-gene cellobiose-family phosphotransferase system (PTS) cluster in CC4, whose essential role in neurovirulence and placental virulence, confirmed in murine models, prompted its designation as LIPI-4 (Maury et al., 2016). While initially considered exclusive to the hypervirulent CC4 (Maury et al., 2016), subsequent studies identified LIPI-4 in sequence type (ST) 382, CC87, including ST87 and ST1459, and CC619 (ST619 and ST2835) (Wang et al., 2019; Lee et al., 2023; Hong et al., 2024; Liu et al., 2024b; Wang et al., 2024). Previous studies have shown that deletion of LIPI-4 in the LM928 (ST87) strain results in reduced capacity for adhesion, invasion, intracellular proliferation, and cell-to-cell spread (Liu et al., 2025a). Notably, at 28 °C, the motility of the ΔLIPI-4 mutant is significantly impaired, with a 35.7-fold downregulation of *flaA* expression. Transmission electron microscopy analysis confirmed the absence of flagellar structures in the ΔLIPI-4 strain, indicating defective flagellar assembly (Liu et al., 2025a). Furthermore, comparative transcriptomic profiling of LM928 and ΔLIPI-4 at 28 °C and 37 °C revealed that the deletion of LIPI-4 substantially alters the expression of genes involved in microbial metabolism in diverse environments and carbon metabolism. Remarkably, transcriptome data also demonstrated a marked upregulation of the *lmo0180* gene in the ΔLIPI-4 strain, with transcription levels increased by 290.02-fold and 32.00-fold at 28 °C and 37 °C, respectively (Liu et al., 2025a).

*lmo0180* is part of the seven-gene operon *lmo0178*–*lmo0184*. Specifically, *lmo0178* encodes a xylose repressor; *lmo0179* and *lmo0180* encode sugar ABC transporter permease proteins; *lmo0181* encodes the corresponding substrate-binding protein; *lmo0182* and *lmo0183* encode α-glucosidase; and *lmo0184* encodes oligo-1,6-glucosidase. The *lmo0179*–*lmo0184* locus forms an ABC transporter system responsible for sugar transport and metabolism. This operon genes belong to the PrfA-repressed Group II category, whose expression is steadily inhibited by PrfA independent of environmental factors such as activated charcoal and cellobiose (Milohanic et al., 2003). By comparison, PrfA exerts positive regulation on ten Group I virulence genes, which encode core virulence factors, including PrfA itself, listeriolysin O (LLO, encoded by *hly*), metalloprotease (Mpl), actin assembly-inducing protein (ActA), phospholipases PlcA and PlcB, internalins InlA, InlB, and InlC, as well as the hexose phosphate transporter (Hpt) (Milohanic et al., 2003).

*L. monocytogenes* employs a well-established intracellular infection strategy involving multiple stages of host interaction. First, bacterial adhesion and invasion are mediated primarily by internalins InlA and InlB. Second, it disrupts the primary or secondary phagosomal membrane via pore-forming and hydrolytic factors including LLO, the Mpl (which activates PlcB), and phospholipases PlcA and PlcB, thereby escaping into the cytoplasm. Once in the cytosol, *L. monocytogenes* utilizes host nutrients to replicate, and then recruits host actin to form actin comet tails. InlC facilitates the reduction of cortical plasma membrane tension, enabling the bacterium to generate protrusions and invade adjacent cells, completing the intracellular infection cycle. After entering neighboring cells, *L. monocytogenes* again disrupts the secondary double-membrane vacuole with LLO, PlcA, and PlcB, initiating a new round of infection (Pizarro-Cerdá and Cossart, 2018; Jia et al., 2026). The coordinated action of the above virulence factors allows *L. monocytogenes* to cross the intestinal, blood–brain, and placental barriers, resulting in severe infections such as sepsis, meningitis, and spontaneous abortion in pregnant women. Our previous studies have revealed significant upregulation of *lmo0180* in LIPI-4-deficient strains, with the highest fold change among all differentially expressed genes (Liu et al., 2025a), implying abnormal gene expression. As a target negatively regulated by the key virulence regulator PrfA, altered expression of *lmo0180* may directly contribute to shifts in *L. monocytogenes* virulence.

This study aimed to investigate the roles of LIPI-4 and the associated gene *lmo0180* in flagellar assembly, biofilm formation, and virulence of *L. monocytogenes*. To this end, we constructed the *lmo0180* single-deletion strain and LIPI-4/*lmo0180* double-deletion strain, and systematically compared their motility capacity, flagellar assembly, and virulence phenotypes with those of the wild-type and ΔLIPI-4 strains. These findings provide insights into the LIPI-4-mediated pathogenic mechanisms in *L. monocytogenes*.

## 2 Materials and methods

### 2.1 Strains and cells

Wild-type *L. monocytogenes* LM928 and the ΔLIPI-4 gene deletion mutant derived from it are long-term preserved in our laboratory. The homozygous deletion mutants (Δ*lmo0180* and ΔLIPI-4/*lmo0180*) and the complementary mutant (CΔ*lmo0180*) were constructed following a previously published method (Liu et al., 2024a; Shi et al., 2024; Liu et al., 2025a). The primers used are provided in **Table 1**. In all experiments, *L. monocytogenes* strains were resuscitated from -80 °C glycerol storage onto Brain Heart Infusion (BHI) agar, and then cultured in BHI broth (Hopebio, Qingdao, China) at 28 or 37 °C with agitation.

**Table 1:** Polymerase chain reaction primers used in this experiment.

Primers	Sequence (5'→3')	Product length (bp)	Target gene
*lmo0180*-F1	AACTGCAGTAATGATTCTTGGCCTAGCGT(PstI)	628	Upstream sequence of the *lmo0180* gene
*lmo0180*-R1	CCTTTTTTGTTGGTTTTTAGTAATTGGACCACCT		
*lmo0180*-F2	AGGTGGTCCAATTACTAAAAACCAACAAAAAAGG	557	Downstream sequence of the *lmo0180* gene
*lmo0180*-R2	CGGAATTCGCCGCTTCTTCGTCTTT (EcoRI)		
Δ*lmo0180* V-F	TTATTCCAAGTGCCAATCTAT	2635/1786	Verify primers of *lmo0180* deletion
Δ*lmo0180* V-R	GAAGCATTGCGATTTGAC		
*lmo0180* C-F	AACTGCAGATGAAAACAGAAAAAAGTGG (Pst I)	849	*lmo0180* gene
*lmo0180* C-R	CCGCTCGAGTTATTCTTTCATTCCGCTCATTGC (Xho I)		
ΔLIPI-4 V-F	GCGATTATTACGGCACTTGC	7061/1051	Verify primers of LIPI-4 deletion
ΔLIPI-4 V-R	TTAGTTTATGGCGATGTCG		

Mouse macrophages RAW264.7, human colon adenocarcinoma Caco-2, and murine fibroblast cell line NCTC clone 929 (L929) were preserved and cultured in our laboratory. RAW264.7 cells were cultured in Dulbecco’s Modified Eagle Medium (DMEM; Servicebio, G4511, Wuhan, China) supplemented with 10% fetal bovine serum (FBS, Excell Bio, Shanghai, China); Caco-2 cells were cultured in Minimum Essential Medium (MEM; Procell Wuhan, China) supplemented with 20% FBS; L929 cells were cultured in MEM with 10% FBS. All cell lines were grown under 5% CO2 and 37 °C conditions.

### 2.2 Growth analysis in BHI broth

Monoclonal colonies of LM928, ΔLIPI-4, Δ*lmo0180*, CΔ*lmo0180*, and ΔLIPI-4/*lmo0180* were individually inoculated into 2 mL of BHI liquid medium for a 12 h preculture. The bacterial suspensions were adjusted to an OD_600_ of 0.6, then subcultured at a 1:100 ratio into fresh BHI medium and incubated at 37 °C with continuous shaking for 12 h. A full-wavelength microplate reader was used to sample 200 μL of bacterial culture from each group at two-hour intervals for the measurement of OD_600_ values.

### 2.3 Motility assay

Bacterial motility was examined by means of a semi-solid agar plate assay and stab inoculation, based on the earlier reported protocol (Shi et al., 2024; Liu et al., 2025a). Five strains of *L. monocytogenes* were cultured overnight in BHI medium and adjusted to an OD_600_ of 0.6. Two microliters of bacterial suspension from each strain were spotted onto semi-solid agar medium (2% NaCl, 1.0% tryptone, and 0.25% agar), with five replicates per strain, and the plates were incubated at 28 °C or room temperature (RT). The swimming halo diameters were then measured and recorded photographically. A bacterial suspension was inoculated using a sterile puncture needle and then transferred into a test tube containing semi-solid culture medium. Following incubation at 28 °C for 48 h, bacterial motility was evaluated by observing the radial expansion of growth.

### 2.4 Transmission electron microscopy assay

TEM experiments were conducted following previously described procedures with minor modifications (Cheng et al., 2018). A 20 μL aliquot of bacterial suspension, incubated overnight at RT, was transferred onto a copper grid coated with carbon film using a pipette and allowed to stand for 3–5 min. Excess liquid was removed by gently touching the edge of the grid with filter paper. Subsequently, 2% phosphotungstic acid was applied to the grid for staining (1–2 min), after which excess stain was absorbed with filter paper, and the grid was air-dried at room temperature. The prepared grids were examined under TEM (GP2004, Servicebio, Wuhan, China), and images were captured.

### 2.5 Quantitative real-time PCR analysis

qRT-PCR analysis was performed as previously described (Liu et al., 2024a). Monoclonal colonies of LM928, ΔLIPI-4, Δ*lmo0180*, and ΔLIPI-4/*lmo0180* were individually inoculated into 15 mL of BHI liquid medium for a 12 h preculture at 28 °C and 37 °C. The bacterial colonies were harvested by centrifugation at 8,000 rpm for 5 min, followed by a single wash with pre-chilled PBS, to obtain the bacterial pellet. Transfer the bacterial pellet into a mortar, add liquid nitrogen, and grind thoroughly until a fine powder is obtained. Subsequent RNA extraction steps should be carried out according to the manufacturer’s instructions (TransGen Biotech, Beijing, China). Total RNA was reverse transcribed into complementary DNA (cDNA) using the PrimeScript™ RT Reagent Kit with gDNA Eraser (Taraka Bio, Beijing, China). Fluorescence quantitative PCR was performed according to the instructions of UltraSYBR Mixture (Cowin Biotech, Jiangsu, China). The transcriptional levels of the *flaA*, *actA, hly*, *inlA*, *inlB*, *inlC*, *mpl*, *plcA*, *plcB* and *lmo0180* genes were quantified. The housekeeping gene *gyrB* was used as an internal control for normalization. Relative transcript levels were quantified using the 2^−ΔΔCt^ method (Livak and Schmittgen, 2001) and expressed as fold changes relative to the control condition. All primers were adopted from previously published studies (Liu et al., 2025a, 2025).

### 2.6 Biofilm-formation assay

To evaluate the effects of LIPI-4 and *lmo0180* on the biofilm formation capacity of *L. monocytogenes*, a crystal violet assay was performed in 48-well microtiter plates according to a previously described method (Liao et al., 2025) with a few adjustments. Overnight cultures of *L. monocytogenes* strains were adjusted to an OD_600_ of 0.6 with sterile BHI broth. The bacterial suspension was added to fresh sterile medium at a 1:100 ratio. Sterile 48-well polystyrene plates were inoculated with 400 μL of fresh culture from each strain in sextuplicate and incubated for 24 h and 48 h at 28 °C and 37 °C. Subsequently, each well was gently rinsed thrice with 400 μL of sterile PBS, air-dried under ambient conditions, fixed with methanol for 30 min, and then stained with a 0.2% crystal violet solution for 20 min. After adding 400 μL of 95% ethanol to each well to dissolve the biofilm for 30 min, the OD_600_ value of each well was detected by a microplate reader (Power Wave XS2, BioTek, USA).

### 2.7 Adhesion and invasion of *L. monocytogenes* in Caco-2 cells

To assess bacterial invasion and adhesion in Caco-2 cells, the following experimental procedures were performed. Overnight cultures of LM928, ΔLIPI-4, Δ*lmo0180*, CΔ*lmo0180*, and ΔLIPI-4/*lmo0180* strains were incubated at 37 °C for 12 h, washed, and resuspended in 0.01 M PBS. For the adhesion assay, Caco-2 cells were inoculated with bacterial strains at a multiplicity of infection (MOI) of 10 and incubated for 1 h. The culture medium was then removed, and the cells were washed twice with PBS. 0.2% Triton X-100 was added to each well. The remaining infected cells were further incubated in complete medium supplemented with gentamicin (100 µg/mL) at 37 °C for 1 h to eliminate residual extracellular bacteria, followed by two washes with PBS and lysis with 0.2% Triton X-100. This was conducted to determine the cell invasion rate of bacteria. The bacterial suspensions were subsequently subjected to serial dilution and plated onto BHI agar plates for CFU enumeration. Adhesion and invasion were quantified as the ratio of the number of colonies remaining after PBS washing to the number of colonies initially inoculated (Chen et al., 2023).

### 2.8 Proliferation of *L. monocytogenes* in RAW 264.7 macrophages

According to the protocol mentioned above, RAW264.7 cells were infected with these bacterial strains at an MOI of 1, followed by a 1 h incubation period. After two washes with PBS, the culture medium was replaced with fresh cell medium containing 100 µg/mL gentamicin to eliminate extracellular bacteria, and this time point was designated as 0 h for the intracellular proliferation assay. After 1 h, the medium was replaced with fresh cell culture medium supplemented with 10 μg/mL gentamicin. At predetermined time points (4, 8, and 12 h post-infection), the cells were washed with PBS, and 1 mL of PBS supplemented with 0.2% Triton X-100 was added to each well to lyse the cells. Bacterial suspensions were subsequently prepared through 10-fold serial dilutions and plated onto BHI agar plates for colony enumeration.

### 2.9 Plaque forming assay

When L929 cells cultured in 6-well plates reached 95% confluency, they were inoculated with bacterial suspension at an MOI of 0.1:1. Subsequent experiments were conducted as previously described (Liu et al., 2025a). After acquiring images of the plaques, 4 mL of 95% anhydrous ethanol was added, and the mixture was incubated for 30 min to achieve complete dissolution. The solution was then thoroughly mixed by repeated pipetting, and a 200 μL aliquot of the resulting suspension was transferred for OD_600_ measurement.

### 2.10 Virulence of *L. monocytogenes* in the mouse model

Six- to eight-week-old Kunming mice were purchased from the Animal Experimental Center of Shihezi University, China. The mice were female Kunming strain with no genetic modifications or genotype variations, and were specific pathogen-free (SPF) grade with normal immune status and good health. Following overnight incubation at 37 °C, bacterial cultures of all strains were adjusted to an OD_600_ of 0.6, then centrifuged to prepare bacterial suspensions in PBS. For the determination of bacterial loads in mouse blood and organs, SPF Kunming mice (n=36) were randomly divided into five experimental groups (6 in each group) and one control group (n=6) using a computer-based random order generator. Before infection, each mouse was individually labeled and weighed. Subsequently, a bacterial suspension diluted to 10^7^ CFU (colony-forming units)/mL was administered via intraperitoneal injection at a volume of 0.2 mL per animal. 48 h after infection, the mice were weighed, and blood samples were collected via orbital sinus puncture. A 100 μL aliquot of each blood sample was then inoculated onto solid BHI agar medium. Mice were sacrificed by cervical dislocation, and livers, spleens, and brains were aseptically excised. The number of CFU was assessed by plating serially diluted organ homogenates onto BHI agar plates, followed by incubation at 37 °C.

### 2.11 Observation of pathological changes

Liver and spleen tissues from different mouse groups were permeabilized and fixed using 4% paraformaldehyde (Servicebio, Wuhan, China). Organ samples were embedded in paraffin blocks, sectioned at a thickness of 3-4 μm, and stained with hematoxylin and eosin (HE) for histopathological examination. The tissue specimens were subsequently examined under a light microscope, and representative pathological findings were documented through photomicrography and descriptive recording.

### 2.12 Statistical analysis

All data were expressed as mean ± Standard Error of the Mean (SEM) and analyzed using the Statistical Package for Social Sciences (SPSS) version 20.0 (SPSS Inc., Chicago, IL, USA) and GraphPad Prism software (version 9.5). Specifically, data were subjected to one-way ANOVA, followed by *post hoc* comparisons using the Least Significant Difference (LSD) and Duncan’s tests. In figures, significant differences (P < 0.05) are denoted by different lowercase letters, while shared letters indicate no significant difference.

## 3 Results

### 3.1 *lmo0180* deletion does not impair the growth of *L. monocytogenes* in BHI broth

**Figure 1:**
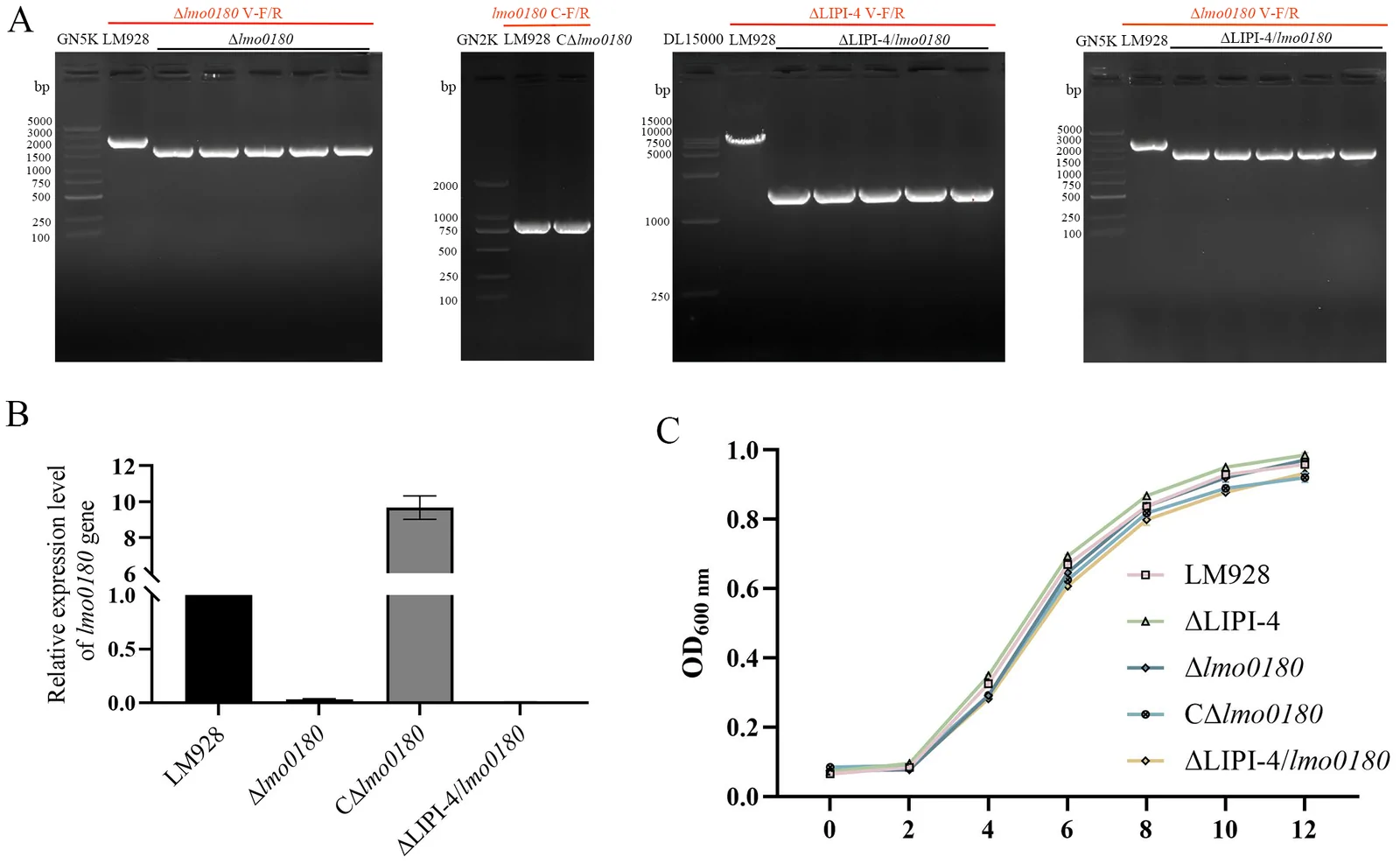
Construction of the deletion mutant strains and complementation strain. **(A)** Identification of Δ*lmo0180*, CΔ*lmo0180*, and ΔLIPI-4/*lmo0180* strains using PCR. **(B)** Identification of Δ*lmo0180*, CΔ*lmo0180*, and ΔLIPI-4/*lmo0180* strains by qRT-PCR. **(C)** The growth curves of *L. monocytogenes* at 37 °C.

The Δ*lmo0180* and ΔLIPI-4/*lmo0180* deletion mutants, together with the CΔ*lmo0180* complementation strain, were successfully constructed and validated by means of PCR and qRT-PCR analyses (**Figures 1A, B**). Growth analysis of the LM928, ΔLIPI-4, Δ*lmo0180*, CΔ*lmo0180*, and ΔLIPI-4/*lmo0180* strains cultured in BHI medium at 37 °C revealed no significant differences in growth kinetics between the Δ*lmo0180*, ΔLIPI-4/*lmo0180*, and CΔ*lmo0180* strains and the wild-type LM928 or ΔLIPI-4 strains. These findings indicate that deletion of the *lmo0180* gene alone, or simultaneous deletion of both LIPI-4 and *lmo0180* genes, does not compromise the normal growth capacity of *L. monocytogenes* in BHI medium under 37 °C conditions (**Figure 1C**).

### 3.2 Deletion of *lmo0180* partially restores flagellar assembly and motility in the ΔLIPI-4 background

**Figure 2:**
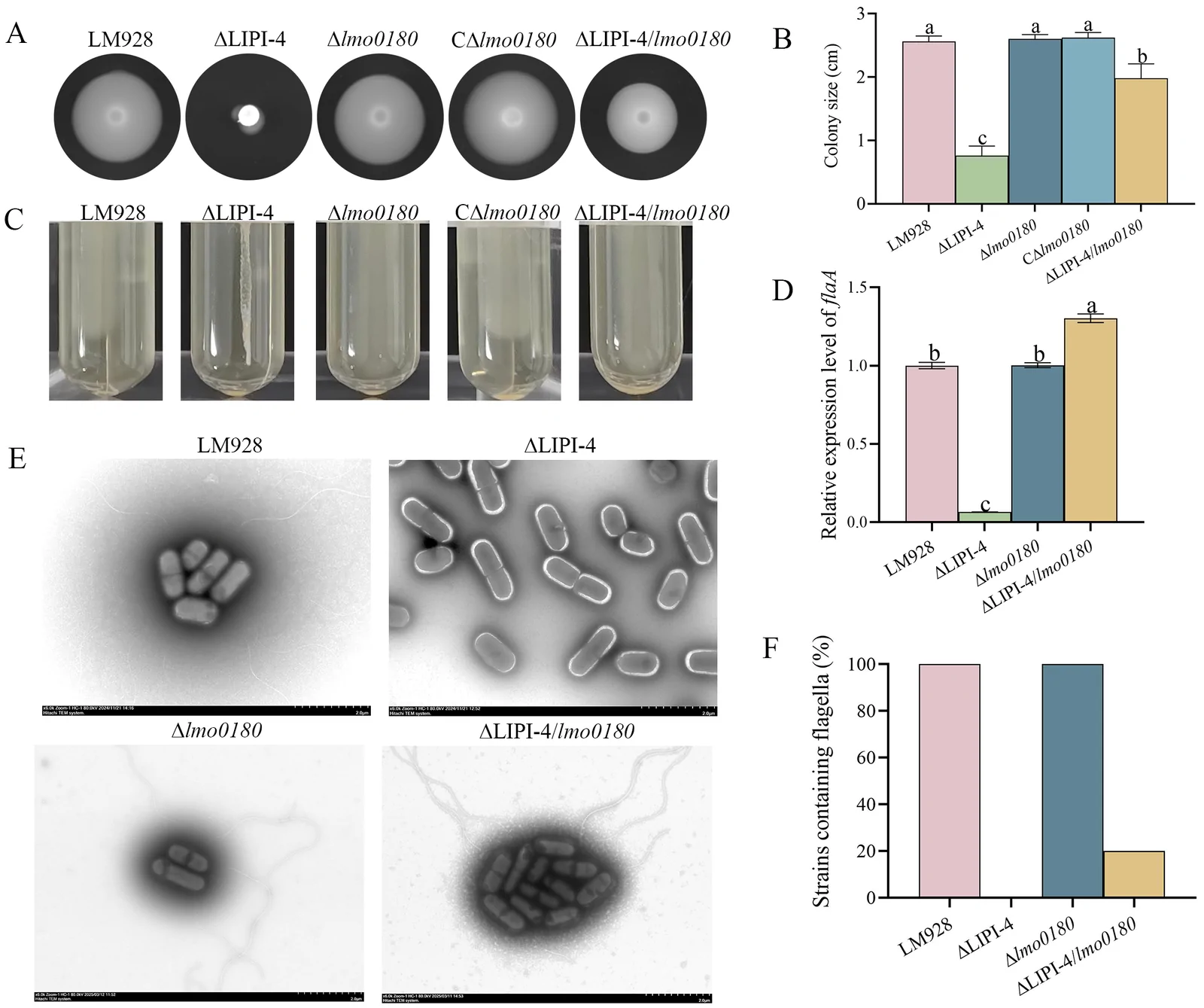
Simultaneous deletion of LIPI-4 and *lmo0180* genes restore flagellar formation in *L. monocytogenes*. **(A)** Swarming ability of LM928, ΔLIPI-4, Δ*lmo0180*, CΔ*lmo0180*, and ΔLIPI-4/*lmo0180* strains at 28 °C. **(B)** The motility zone diameters of each strain were measured and statistically analyzed. **(C)** Puncture tests of LM928, ΔLIPI-4, Δ*lmo0180*, CΔ*lmo0180*, and ΔLIPI-4/*lmo0180* strains in a semisolid medium at 28 °C. **(D)** Changes in the expression levels of the *flaA* gene among the LM928, ΔLIPI-4, Δ*lmo0180*, and ΔLIPI-4/*lmo0180* strains. **(E)** Flagella Production in LM928, ΔLIPI-4, Δ*lmo0180*, and ΔLIPI-4/*lmo0180* Strains at RT as Observed by Transmission Electron Microscopy (TEM). **(F) **Percentage of bacteria carrying at least one flagellum, as observed under transmission electron microscopy, for strains LM928, ΔLIPI-4, Δ*lmo0180*, and ΔLIPI-4/*lmo0180*. Different lowercase letters in the figure indicate significant differences among treatments by ANOVA followed by Duncan’s multiple range test (P < 0.05).

*L. monocytogenes* strains are highly flagellated and motile at low temperatures, 30 °C and below, and are typically not motile at temperatures of 37 °C or above (Gründling et al., 2004). Consequently, the motility of various *L. monocytogenes* strains was evaluated at 28 °C. Comparative analysis of motility phenotypes revealed a significant reduction in the swimming halo diameter of the ΔLIPI-4 strain compared to the wild-type LM928 (P<0.05). In contrast, the Δ*lmo0180* and CΔ*lmo0180* strains exhibited swimming halos that did not differ significantly from those of LM928 (P>0.05). Notably, the ΔLIPI-4/*lmo0180* strain displayed partial restoration of *L. monocytogenes* motility, although its swimming halos remained smaller than those of both LM928 and the Δ*lmo0180* strain (P<0.05) (**Figures 2A, B**). Consistent with these findings, **Figure 2C** illustrates that the radial spreading patterns of the Δ*lmo0180* and CΔ*lmo0180* strains were comparable to those of the wild-type control in stab inoculation, whereas the ΔLIPI-4 strain exhibited impaired expansion. The ΔLIPI-4/*lmo0180* strain regained the capacity for peripheral diffusion under the same conditions. Subsequently, the transcriptional levels of the *flaA* gene were quantified in LM928, ΔLIPI-4, Δ*lmo0180*, and ΔLIPI-4/*lmo0180* strains cultured at 28 °C using qRT-PCR. The results showed a 15.4-fold decrease in *flaA* transcription in the ΔLIPI-4 strain relative to LM928 (P<0.05), whereas no significant change was detected in the Δ*lmo0180* strain (P>0.05). In contrast, the ΔLIPI-4/*lmo0180* strain exhibited a 1.3-fold upregulation in *flaA* gene expression (P<0.05) (**Figure 2D**).

To investigate flagellar assembly in *L. monocytogenes* strains, TEM was used to examine bacterial cultures incubated at 25-28 °C. Consistent with expected phenotypic characteristics, flagella were clearly observed in both the LM928 and Δ*lmo0180* strains, whereas no flagellar structures were detected in the ΔLIPI-4 strain. Notably, partial restoration of flagellar assembly was observed in the ΔLIPI-4/*lmo0180* strain (**Figure 2E**). Among evaluable cells, flagella-bearing cells were detected in LM928 (44/44), ΔLIPI-4 (0/134), Δ*lmo0180* (11/11), and ΔLIPI-4/*lmo0180* (2/5) (**Figure 2F**).

### 3.3 LIPI-4 negatively regulates biofilm formation in *L. monocytogenes*

**Figure 3:**
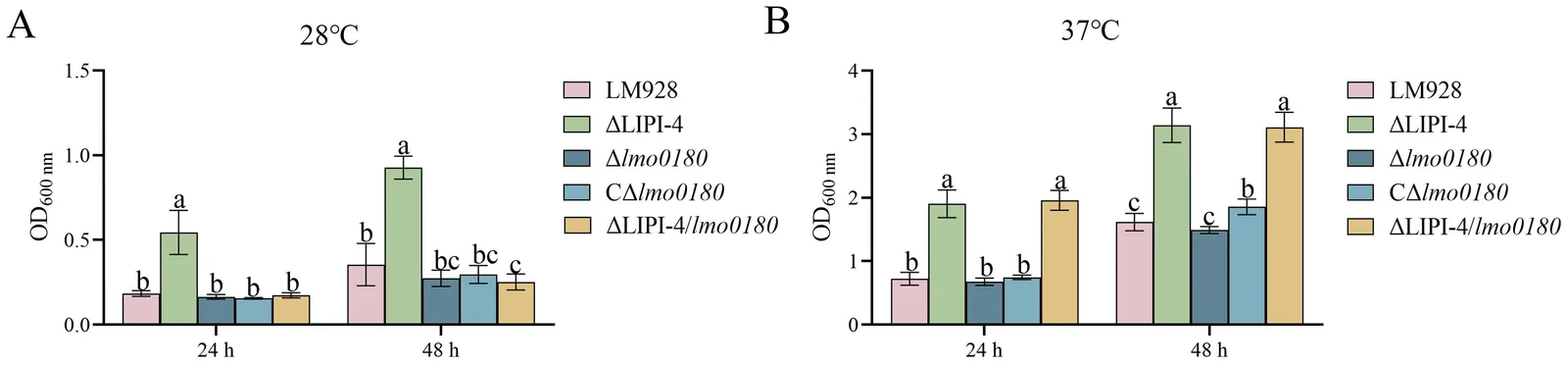
Determination of the biofilm formation capacity of five *L. monocytogenes* strains. Biofilm formation by the LM928, ΔLIPI-4, Δ*lmo0180*, CΔ*lmo0180*, and ΔLIPI-4/*lmo0180* strains was assessed at 28 °C **(A)** and 37 °C **(B)**, respectively, with eight replicate measurements per strain. Different lowercase letters in the figure indicate significant differences among treatments by ANOVA followed by Duncan’s multiple range test (P < 0.05).

The biofilm formation ability of *L. monocytogenes* strains LM928, ΔLIPI-4, Δ*lmo0180*, CΔ*lmo0180* and ΔLIPI-4/*lmo0180* was assessed using crystal violet staining. At 28 °C, biofilm formation by the ΔLIPI-4 strain was significantly higher than that of the other strains at both 24 h and 48 h (P<0.05). No significant differences in biofilm formation were observed among the other strains at 24 h (P>0.05). At 48 h, the biofilm formation of the ΔLIPI-4/*lmo0180* strain was slightly lower than that of the LM928 strain (P<0.05), with no significant difference among the LM928, Δ*lmo0180*, and CΔ*lmo0180* strains (P>0.05) (**Figure 3A**). At 37 °C, both the ΔLIPI-4 and ΔLIPI-4/*lmo0180* strains exhibited significantly higher biofilm formation compared to the LM928, Δ*lmo0180*, and CΔ*lmo0180* strains (P<0.05). At 24 h, no significant differences were detected among the LM928, Δ*lmo0180*, and CΔ*lmo0180* strains (P>0.05). By 48 h, biofilm formation in the CΔ*lmo0180* strain was slightly higher than that of the LM928 and Δ*lmo0180* strains (P<0.05) (**Figure 3B**). Overall, LIPI-4 negatively regulates biofilm formation in *L. monocytogenes*.

### 3.4 Contributions of LIPI-4 and *lmo0180* to host cell adhesion, invasion, intracellular growth and intercellular spread

**Figure 4:**
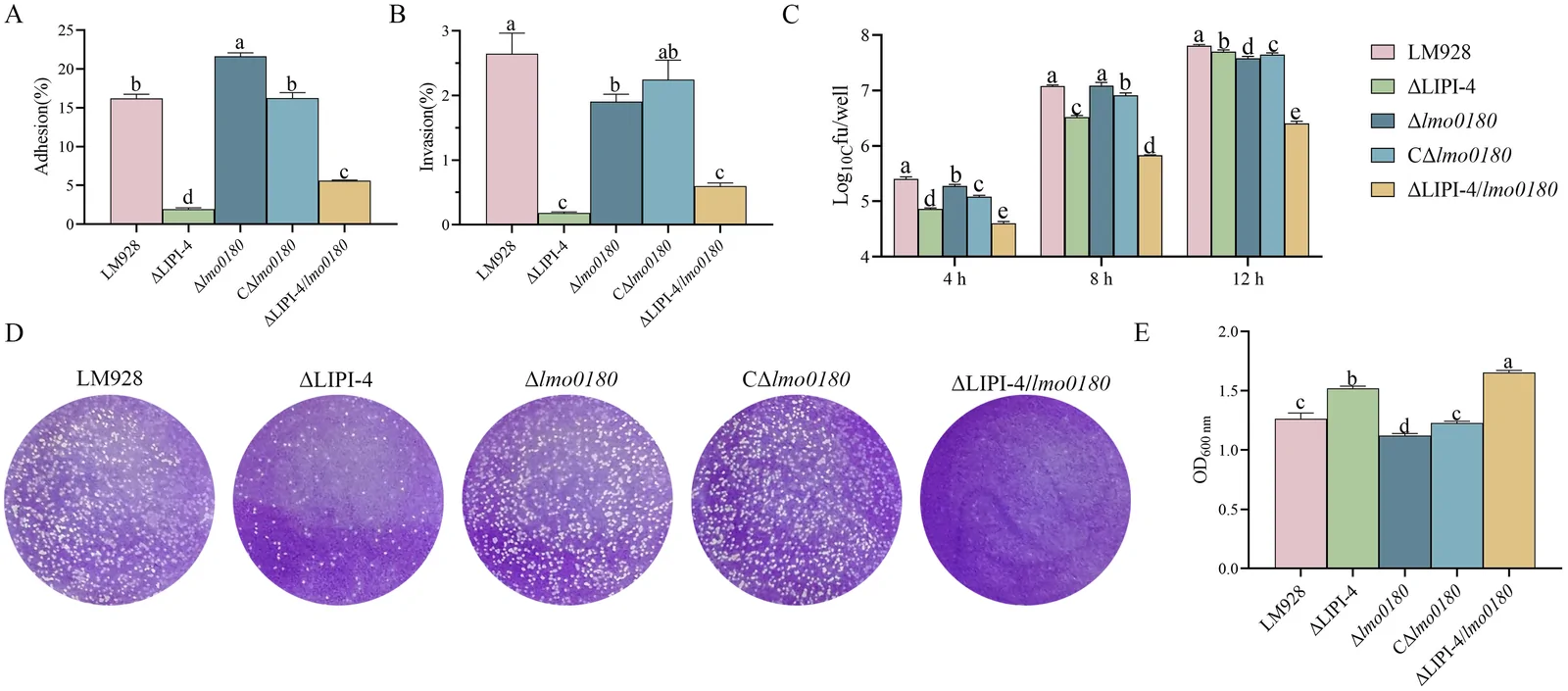
Role of LIPI-4 and *lmo0180* gene in host cell challenge by *L. monocytogenes*. Adhesion **(A)** and invasion **(B)** capacities of the LM928, ΔLIPI-4, Δ*lmo0180*, CΔ*lmo0180*, and ΔLIPI-4/*lmo0180* strains in Caco-2 Cells. **(C)** The intracellular proliferation capacity of five *L. monocytogenes* strains in RAW264.7 cells was assessed. **(D)** Comparison of plaque-forming abilities in L929 cells infected with five *L. monocytogenes* strains. **(E)** The plaque-forming capacity of each strain was quantified by measuring the OD_600_ value. A lower OD_600_ value corresponds to a higher number of plaques formed. Different lowercase letters in the figure indicate significant differences among treatments by ANOVA followed by Duncan’s multiple range test (P < 0.05).

To investigate the roles of LIPI-4 and *lmo0180* in bacterial adhesion and invasion, Caco-2 epithelial cells were used to compare the adhesion and invasion capacities of the ΔLIPI-4, *lmo0180*, CΔ*lmo0180*, and ΔLIPI-4/*lmo0180* strains with those of the LM928 strain. The results indicate that, compared to the wild-type strain, the Δ*lmo0180* strain exhibited a significantly increased adhesion rate, whereas the ΔLIPI-4 and ΔLIPI-4/*lmo0180* strains showed a marked reduction in adhesive capacity, with no significant difference observed for the CΔ*lmo0180* strain (**Figure 4A**). In comparison with the ΔLIPI-4/*lmo0180* strain, the ΔLIPI-4 strain demonstrated reduced adhesive ability, while the Δ*lmo0180* and CΔ*lmo0180* strains exhibited significantly enhanced adhesion. With respect to invasion capacity, the ΔLIPI-4, Δ*lmo0180*, and ΔLIPI-4/*lmo0180* strains all displayed significantly decreased invasive potential relative to the wild-type strain, whereas the CΔ*lmo0180* strain showed no notable difference. Compared to the ΔLIPI-4/*lmo0180* strain, the ΔLIPI-4 strain exhibited diminished invasiveness, while the Δ*lmo0180* strain demonstrated a significant increase in invasion capacity (**Figure 4B**).

The intracellular multiplication of LM928, ΔLIPI-4, Δ*lmo0180*, CΔ*lmo0180*, and ΔLIPI-4/*lmo0180* strains was analyzed after internalization into RAW264.7 cells to investigate the role of LIPI-4 and *lmo0180* in additional phases of the cell infection process. The results indicate that the intracellular proliferation of all bacterial strains increased over time. Except for the CΔ*lmo0180* strain, which showed no significant difference in intracellular bacterial load at 8 h, the proliferation levels of the remaining strains were significantly lower than that of LM928 at 4, 8, and 12 h. Compared to the ΔLIPI-4 and Δ*lmo0180* strains, the ΔLIPI-4/*lmo0180* strain exhibited a considerably reduced intracellular bacterial count. Furthermore, it was observed that the intracellular proliferation of ΔLIPI-4 gradually approached that of LM928 over time, whereas the intracellular bacterial load of ΔLIPI-4/*lmo0180* remained consistently lower than those of LM928, ΔLIPI-4, and Δ*lmo0180* strains (**Figure 4C**).

The ability of various *L. monocytogenes* strains to spread from cell to cell was evaluated using L929 cells. As illustrated in **Figure 4D**, the ΔLIPI-4 and ΔLIPI-4/*lmo0180* mutants exhibited significantly reduced plaque formation compared to the LM928 strain. Additionally, the ΔLIPI-4/*lmo0180* double mutant produced fewer plaques than the Δ*lmo0180* or ΔLIPI-4 single mutants. Simultaneously measuring the OD_600_ value, which was negatively correlated with plaque formation ability, revealed that the *lmo0180* mutant formed more plaques than the LM928 strain. The remaining experimental results are consistent with the observations shown in **Figure 4E**.

In summary, a single deletion of the *lmo0180* gene has little effect on the adhesion, invasion, intracellular proliferation, and intercellular spread of *L. monocytogenes*; however, the coordinated regulation of the LIPI-4 and *lmo0180* genes plays a significant role in enhancing intracellular proliferation and cell-to-cell spread capabilities.

### 3.5 Double deletion of LIPI-4 and *lmo0180* severely impairs *L. monocytogenes* virulence in mice

**Figure 5:**
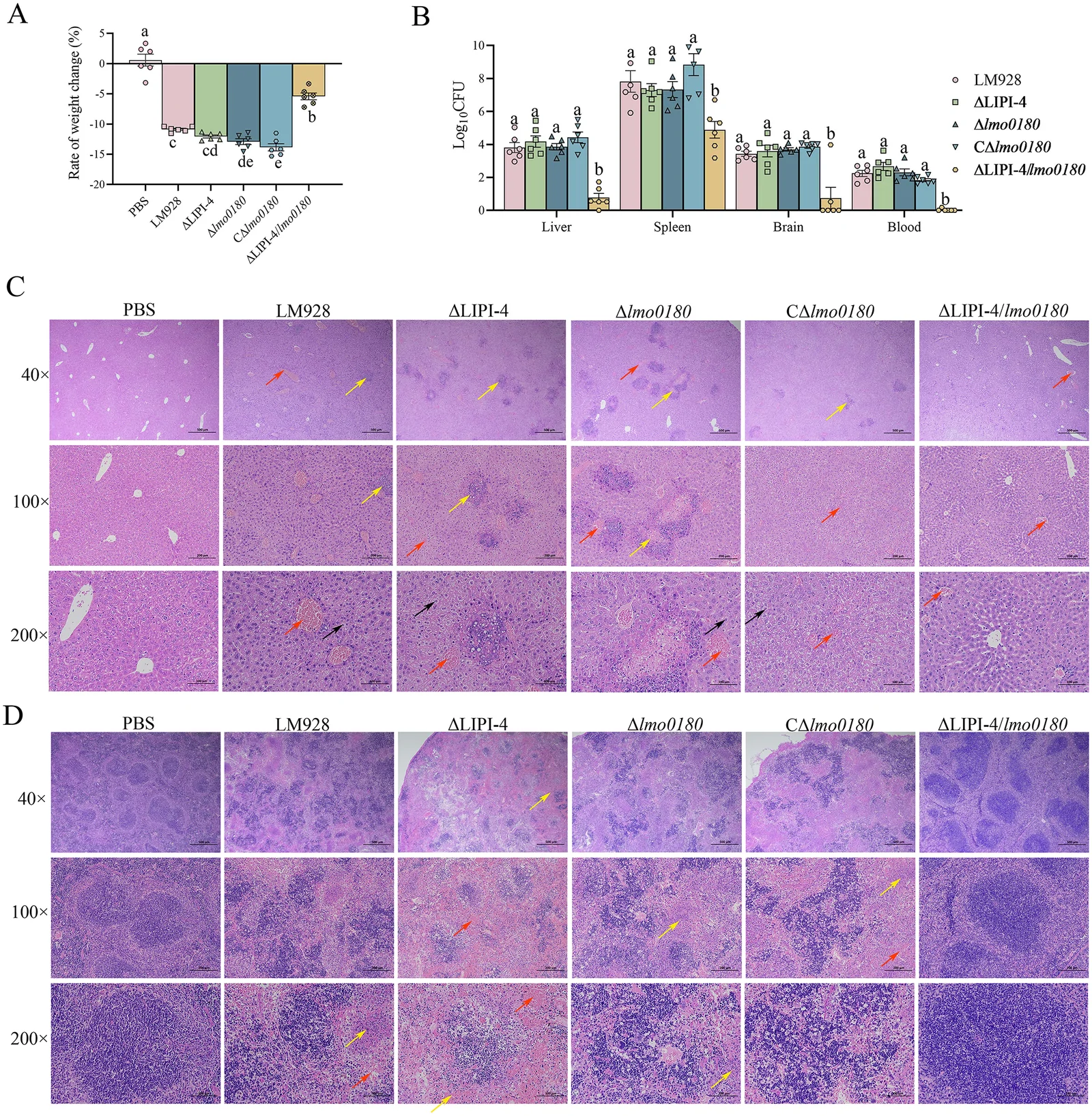
Simultaneous deletion of the LIPI-4 and *lmo0180* genes reduce the colonization ability of *L. monocytogenes* in mice and alleviates pathological damage. **(A)** Body weight change rate in mice 48 h after infection with *L. monocytogenes*. **(B)** Bacterial counts were monitored in the liver, spleen, brain, and blood of mice at 48 h post-challenge. The livers **(C)** and spleens **(D)** of mice infected with LM928, ΔLIPI-4, Δ*lmo0180*, CΔ*lmo0180*, and ΔLIPI-4/*lmo0180* were harvested (Red arrows indicate hemorrhage and hyperemia; yellow arrows indicate necrotic foci; black arrows indicate hepatocellular swelling); the tissues were fixed, and hematoxylin-eosin sections were made to observe the pathological changes. Different lowercase letters in the figure indicate significant differences among treatments by ANOVA followed by Duncan’s multiple range test (P < 0.05).

To investigate the impact of the LIPI-4 and *lmo0180* deletions on the *in vivo* colonization capacity of *L. monocytogenes*, mice were infected with LM928, ΔLIPI-4, Δ*lmo0180*, CΔ*lmo0180*, and ΔLIPI-4/*lmo0180*. 48 h after intraperitoneal injection, the body weight of all five groups of mice infected with *L. monocytogenes* decreased significantly compared to the PBS group. The ΔLIPI-4/*lmo0180* group exhibited the least body weight loss (5.41%), while the LM928, ΔLIPI-4, Δ*lmo0180*, and CΔ*lmo0180* groups exhibited body weight reduction rates of 10.86%, 12.05%, 12.89%, and 15.30%, respectively (**Figure 5A**). In the liver, spleen, brain, and blood, the bacterial loads of the LM928, ΔLIPI-4, Δ*lmo0180*, and CΔ*lmo0180* groups showed no significant differences among them (P>0.05). However, the ΔLIPI-4/*lmo0180* group exhibited a significantly lower bacterial load in the blood and all organs compared to the other groups (P<0.05) (**Figure 5B**).

Next, histopathological examinations of the spleen and liver were performed in mice to evaluate the tissue damage induced by *L. monocytogenes*. As demonstrated by histopathological evaluation of liver tissue, considerable disparities in the extent of liver tissue damage were evident among the experimental groups. The PBS control group exhibited normal structure, with no lesions. By contrast, the LM928 and CΔ*lmo0180* groups demonstrated hepatocyte swelling and scattered small necrotic foci. The most severe damage was observed in the ΔLIPI-4 and Δ*lmo0180* groups, characterized by destruction of hepatic lobule structure and multiple necrotic foci. The ΔLIPI-4/*lmo0180* group exhibited only mild inflammation and congestion (**Figure 5C**). Splenic histopathological analysis revealed that, in comparison with the PBS group, the ΔLIPI-4/*lmo0180* group exhibited normal splenic architecture. The LM928 group exhibited enlarged germinal centers and multifocal necrosis. In contrast, the CΔ*lmo0180* group demonstrated mild structural disorganization and focal necrosis. By contrast, the Δ*lmo0180* and ΔLIPI-4 groups exhibited destruction of white pulp architecture, massive lymphocyte apoptosis, and extensive coagulative necrosis in the red pulp (**Figure 5D**). The results indicate that the deletion of either the LIPI-4 or *lmo0180* gene does not reduce the virulence of *L. monocytogenes*; in fact, it may even slightly increase its virulence. However, simultaneous deletion of both the LIPI-4 and *lmo0180* genes has been shown to reduce the virulence of *L. monocytogenes* significantly.

### 3.6 LIPI-4 and *lmo0180* modulate the transcription of core virulence genes of *L. monocytogenes* in BHI medium

**Figure 6:**
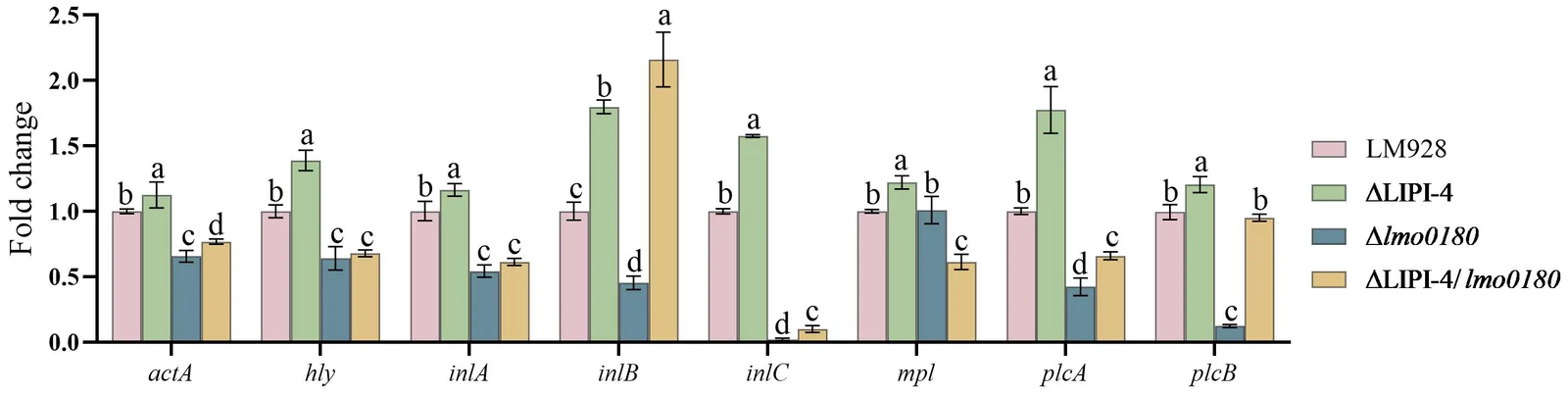
Transcription levels of virulence genes in *L. monocytogenes* strains. qRT-PCR was performed on cDNA templates from LM928, ΔLIPI-4, Δ*lmo0180*, and ΔLIPI-4/*lmo0180* strains harvested 12–14 h after incubation at 37 °C to determine the fold change of virulence genes.

We analyzed the expression levels of virulence genes *actA*, *hly*, *inlA*, *inlB*, *inlC*, *mpl*, *plcA*, and *plcB* in *L. monocytogenes* strains LM928, ΔLIPI-4, Δ*lmo0180*, and ΔLIPI-4/*lmo0180* cultured in BHI medium at 37 °C. Compared with LM928, the transcription levels of all virulence genes were significantly increased in the ΔLIPI-4 strain (P < 0.05). In the Δ*lmo0180* strain, all tested genes were markedly downregulated (P < 0.05) except for *mpl*, which showed no significant difference (P > 0.05). For the ΔLIPI-4/*lmo0180* strain, the transcription level of *inlB* was significantly elevated (P < 0.05), *plcB* had no significant change (P > 0.05), and *actA*, *hly*, *inlA*, *inlC*, *mpl*, and *plcA* genes exhibited significantly decreased transcription levels (P < 0.05) (**Figure 6**).

## 4 Discussion

Although LIPI-4 was identified in 2016 as a virulence gene cluster conferring neural and placental tropism in mouse model (Maury et al., 2016), subsequent studies have largely been confined to epidemiological surveys (Wang et al., 2019; Lee et al., 2023; Hong et al., 2024; Liu et al., 2024b; Wang et al., 2024; Barcellos Jaskulski et al., 2026; Lee et al., 2026), its molecular mechanism remains largely unknown. Our previous work demonstrated that the ΔLIPI-4 mutant was non-motile and failed to produce flagella at 28 °C, despite no significant transcriptional changes in canonical flagellar genes except for *flaA*, suggesting that LIPI-4 regulates flagellar biogenesis through an unconventional pathway (Liu et al., 2025a). Extensive mutational analyses further showed that this phenotype could not be attributed to disruption of any individual LIPI-4 gene or gene subset, indicating that the integrity of the pathogenicity island, rather than a single component, is required. Guided by comparative transcriptomic analyses, which identified marked upregulation of *lmo0180* in the ΔLIPI-4 mutant, the present study functionally evaluated the contribution of *lmo0180*. Importantly, although differential expression alone does not imply causality, genetic analysis demonstrated that deletion of *lmo0180* partially rescued the flagellar defects caused by LIPI-4 deletion, revealing a previously unrecognized genetic interaction between these loci.

*L. monocytogenes* can successfully contaminate processed foods due to its ability to persist for extended periods as biofilms on food processing surfaces (Voloshchuk et al., 2025). Previous studies have reported conflicting relationships between flagellar motility and biofilm formation in *L. monocytogenes*. While intact flagella facilitate initial surface attachment and biofilm development in many strains (Lemon et al., 2007; Gueriri et al., 2008), loss of motility has also been associated with hyperbiofilm phenotypes under specific genetic backgrounds (Todhanakasem and Young, 2008; Yu et al., 2023). These discrepancies suggest that the relationship between flagellar assembly and biofilm formation is highly context-dependent. Our findings support this view. The biofilm-forming capacity of *L. monocytogenes* strains was evaluated at 28 °C and 37 °C. Notably, the ΔLIPI-4 strain exhibited greater biofilm-forming ability than the LM928 strain under both conditions. Furthermore, at 28 °C, the ΔLIPI-4/*lmo0180* strain displayed a biofilm phenotype comparable to those of LM928 and the Δ*lmo0180* mutant, whereas at 37 °C its biofilm-forming ability remained significantly higher than that of LM928 and the Δ*lmo0180* mutant and was comparable to that of the ΔLIPI-4 mutant. These observations suggest that the relationship between flagellar assembly and biofilm formation is strongly influenced by the disrupted genetic locus, strain background, and environmental conditions. In particular, deletion of LIPI-4 was associated with enhanced biofilm formation, whereas additional deletion of *lmo0180* partially restored the biofilm phenotype at 28 °C but not at 37 °C. This temperature-dependent phenotype suggests that the functional interaction between LIPI-4 and *lmo0180* may be influenced by temperature-dependent regulatory pathways. However, the molecular basis underlying this phenomenon remains unclear and warrants further investigation.

Based on the above phenotypic analyses, we next evaluated the pathogenic potential of the mutant strains. In our previous study, the ΔLIPI-4 mutant displayed reduced host infection capability, which was initially presumed to result from the defect in flagellar assembly (Liu et al., 2025a). However, the *in vitro* infection assays in the present study revealed distinct and more complex phenotypes. Compared with the wild-type strain LM928, the ΔLIPI-4/*lmo0180* double mutant exhibited significantly reduced adhesion to and invasion of Caco-2 cells, although these indices were slightly higher than those of the ΔLIPI-4 single mutant. Notably, intracellular proliferation and cell-to-cell spreading of the ΔLIPI-4/*lmo0180* strain were drastically lower than those of the ΔLIPI-4 strain, indicating a synergistic effect of LIPI-4 and *lmo0180* on infection processes. Consistent with the *in vitro* results, *in vivo* virulence assessment using a mouse model showed that the ΔLIPI-4/*lmo0180* strain caused the mildest body weight loss, the lowest bacterial burdens in the liver, spleen, brain, and blood, and the least histopathological damage among all tested strains. Unexpectedly, however, the ΔLIPI-4 single mutant did not recapitulate the significant virulence attenuation observed in our previous report. No obvious differences in body weight change, bacterial load, or tissue lesions were observed between the ΔLIPI-4 and wild-type strains; indeed, the ΔLIPI-4 strain caused slightly more severe tissue damage than LM928. The Δ*lmo0180* and complementary CΔ*lmo0180* strains behaved similarly to the wild-type strain in all virulence-related indicators, consistent with their lack of notable phenotypic changes in motility and flagellar assembly. Taken together, these findings indicate that LIPI-4 and *lmo0180* genetically interact to influence multiple virulence-associated phenotypes.

We measured the transcription levels of a series of virulence genes positively regulated by PrfA in various mutant strains. The results showed that in the ΔLIPI-4 strain, the transcription levels of all tested virulence genes (8/8) were significantly upregulated. Notably, the expression of *lmo0180* was also significantly increased in this strain, while animal experiments indicated that its virulence was not significantly reduced. One possible explanation is that deletion of LIPI-4 alters the regulatory network associated with PrfA, resulting in coordinated changes in the expression of both *lmo0180* and PrfA-regulated virulence genes. However, whether these transcriptional changes are causally linked remains to be determined.

Flagella are multifunctional organelles that enable bacteria to migrate toward favorable environments. Beyond their roles in motility and chemotaxis, flagella have been implicated in diverse biological processes, including surface adhesion, biofilm formation, secretion of virulence factors, and modulation of host immune responses (Duan et al., 2013). In *L. monocytogenes*, flagellar biosynthesis is tightly regulated in a temperature-dependent manner through the MogR–GmaR regulatory system, which differs fundamentally from the hierarchical regulatory cascade described in many Gram-negative bacteria. At 37 °C, MogR represses flagellar gene expression, rendering the bacterium non-motile, whereas at temperatures of 30 °C or below, GmaR relieves this repression and permits flagellar synthesis and motility (Gründling et al., 2004; Kamp and Higgins, 2011). Although flagella contribute to the virulence of many bacterial pathogens, their role in *L. monocytogenes* remains controversial. Several studies have shown that disruption of flagellar genes, including *flaA*, *fliF*, and *fliI*, abolishes motility and impairs adhesion and invasion of cultured epithelial cells, yet produces little or no reduction in virulence in mouse infection models (Dons et al., 2004; Way et al., 2004; Bigot et al., 2005). These findings suggest that flagella facilitate host cell interaction but are not, by themselves, major determinants of systemic virulence.

Our previous study proposed that the attenuated infection phenotype of the ΔLIPI-4 mutant might be attributable to the loss of flagellar assembly (Liu et al., 2025a). However, the present study provides a more nuanced perspective. Although deletion of *lmo0180* in the ΔLIPI-4 background partially restored flagellar assembly and bacterial motility, the double mutant exhibited markedly reduced adhesion, invasion, intracellular proliferation, cell-to-cell spread, and virulence in the mouse model. These findings indicate that restoration of flagellar assembly alone is insufficient to restore virulence, suggesting that the contribution of LIPI-4 to pathogenicity extends beyond its effects on flagellar biogenesis. Instead, additional LIPI-4-dependent pathways are likely involved in regulating host infection and disease progression.

Although our findings demonstrate that LIPI-4 and *lmo0180* cooperatively influence flagellar motility and virulence in *L. monocytogenes*, the molecular basis underlying this interaction remains unclear. In particular, while deletion of *lmo0180* alone did not affect flagellar assembly or virulence-associated phenotypes, deletion of *lmo0180* in the ΔLIPI-4 background restored flagellar structure, motility, and virulence-related defects. These results suggest a genetic interaction between LIPI-4 and *lmo0180*, but do not establish a direct regulatory relationship between these two loci. One possible explanation is that *lmo0180* may function as a modulator of cellular processes affected by LIPI-4 deficiency, thereby compensating for the loss of LIPI-4-associated functions. However, the precise mechanism by which *lmo0180* deletion restores flagellar assembly and virulence remains to be determined. Future studies combining comparative transcriptomic and proteomic analyses of the single and double mutants, together with complementation experiments, will be necessary to clarify whether *lmo0180* acts through regulation of flagellar-associated pathways, virulence gene expression, or other cellular processes.

## 5 Conclusions

Our study demonstrates that LIPI-4 and *lmo0180* functionally interact to influence flagellar assembly, bacterial motility, and virulence-associated phenotypes in *L. monocytogenes*. Although deletion of *lmo0180* alone had little detectable effect on motility or virulence, deletion of *lmo0180* in the ΔLIPI-4 background partially restored *flaA* expression, flagellar assembly, and bacterial motility. In contrast, the ΔLIPI-4/*lmo0180* double mutant exhibited markedly reduced adhesion, invasion, intracellular proliferation, cell-to-cell spread, and virulence in both cell culture and mouse infection models. In addition, the ΔLIPI-4 mutant showed increased transcription of PrfA-regulated virulence genes, whereas expression of most of these genes was reduced in the ΔLIPI-4/*lmo0180* double mutant. These transcriptional changes may contribute to the attenuated virulence phenotype observed in the double mutant. Collectively, our findings provide new evidence that LIPI-4 and *lmo0180* genetically interact to influence motility and pathogenicity in *L. monocytogenes*, offering new insights into the molecular basis of LIPI-4-associated virulence.

## Data Availability

The original contributions presented in the study are included in the article/supplementary material. Further inquiries can be directed to the corresponding author/s.
