# Supplementary material for: LIPI-4 and lmo0180 cooperatively regulate flagellar motility and virulence in Listeria monocytogenes
Source: Front Cell Infect Microbiol. 2026 Sep 3;16:1915886. doi: 10.3389/fcimb.2026.1915886 (PMC13581670; doi:10.3389/fcimb.2026.1915886)
Supplement: Supplementary file 1 [file Supplementaryfile1.docx]

Catalogue numbers of the ladders:

DNA markers GN2K (Cat. No. G3378-01, Servicebio, Wuhan, China) and GN5K (Cat. No. G3361-01, Servicebio, Wuhan, China) were used. The DL15,000 DNA Marker (Cat. No. 3582Q, TaKaRa, Japan) was also used in this study.
